# Letter to the Editor: Pathogens detected from patients with acute respiratory infections negative for SARS-CoV-2, Saitama, Japan, 2020

**DOI:** 10.5365/wpsar.2024.15.1.1135

**Published:** 2024-02-23

**Authors:** Yuzo Arima, Yuuki Tsuchihashi, Osamu Takahara, Reiko Shimbashi, Takeshi Arashiro, Ayu Kasamatsu, Yusuke Kobayashi, Katsuhiro Komase, Takuri Takahashi, Kanako Otani, Fangyu Yan, Taro Kamigaki, Kiyosu Taniguchi, Motoi Suzuki

**Affiliations:** a Center for Surveillance, Immunization, and Epidemiologic Research, National Institute of Infectious Diseases, Tokyo, Japan.; b Center for Field Epidemic Intelligence, Research and Professional Development, National Institute of Infectious Diseases, Tokyo, Japan.; c Department of Pathology, National Institute of Infectious Diseases, Tokyo, Japan.; d National Hospital Organization Mie National Hospital, Mie, Japan.

Dear Editor,

We read with interest the article by Miyashita et al. (*1*) and commend them for conducting syndromic acute respiratory infection (ARI) surveillance during 2020, a challenging year for surveillance. The COVID-19 pandemic reminded us that the number of cases detected directly relates to testing intensity, and that test data (the number of tests performed) and positivity (the proportion of tests that are positive for a given pathogen or pathogens) should be considered when interpreting trends in surveillance data. (*2*-*5*) The data from Miyashita et al. provide an empirical illustration of the importance of test data.

For instance, when comparing the respiratory pathogen data (excluding SARS-CoV-2 and influenza, as per the study design) among the 10 age groups, test data enable the interpretation of the number of test-positive cases *accounting* for the number of tests performed (Miyashita et al., Table 2). As the authors noted, while those aged 80–89 years had the most tests (*n* = 389 samples), positivity ranked seventh, at only 8.7%; although case detections ranked second (*n* = 30), this was probably the result of more testing and ignoring the test and positivity data would have conveyed a misleading picture. In contrast, those aged 0–9 years had the highest case detections (*n* = 77) *and* positivity (40.5%). Compared to those aged 80–89 years, the paediatric group had more than double the number of detections despite having only half the number of tests (*n* = 192 samples), thus the high detection counts cannot be explained by more testing. The fact that children were most affected is also suggested when restricted to lower respiratory tract infections (Miyashita et al., Table 4, collapsed to three age groups). While 0–14-year-olds had fewer detections (*n* = 39) compared to 15–64-year-olds (*n* = 69) and ≥ 65-year-olds (*n* = 59), they had a substantially higher positivity at 52.0%, 15.9% and 6.9%, respectively. Taken together with the much smaller underlying paediatric population (age distribution of Saitama Prefecture’s 2020 population: (*1*) 11.7% 0–14 years, 62.0% 15–64 years, 26.3% ≥ 65 years), the test data strongly suggest that children were the age group most burdened by respiratory pathogens.

Test data can also help with temporal interpretations of surveillance data. As the authors comment, fewer detections in the latter half of 2020 could be due to a reduced number of samples. Reduced testing in November (*n* = 28) and December (*n* = 9)—combined with high positivity—supports this interpretation, suggesting that ARI surveillance sensitivity may have been lower compared to April, when test frequency was highest (*n* = 521), resulting in more detections but with low positivity (Miyashita et al., Fig. 1). In contrast, during June and September, while there were also fewer tests (*n* = 114) resulting in fewer detections, *positivity* was also at its lowest; less testing alone generally does not lead to lower positivity, and the observed pattern rather suggests a genuine reduction in respiratory pathogen prevalence. Again, all else being equal, accounting for test data allows for more confident assessments of detected case numbers.

To summarize, when comparing across “person,” “time” or “place,” explicitly accounting for testing helps address testing bias and improve data interpretation, in ways not possible with numerator case data alone. (*2*-*5*) Surveillance workers should recognize that appropriate interpretation of data has real public health implications, as surveillance data directly inform situational awareness, risk assessment and response.
